# Microbiological Implications of Periurban Agriculture and Water Reuse in Mexico City

**DOI:** 10.1371/journal.pone.0002305

**Published:** 2008-05-28

**Authors:** Marisa Mazari-Hiriart, Sergio Ponce-de-León, Yolanda López-Vidal, Pilar Islas-Macías, Rosa Isabel Amieva-Fernández, Francisco Quiñones-Falconi

**Affiliations:** 1 Instituto de Ecología, Departamento de Ecología de la Biodiversidad, Universidad Nacional Autónoma de México, Mexico City, Mexico; 2 Instituto Nacional de Ciencias Médicas y Nutrición Salvador Zubirán, Unidad de Epidemiología Clínica, Mexico City, Mexico; 3 Facultad de Medicina, Laboratorio de Inmunología Molecular Microbiana, Universidad Nacional Autónoma de México, Mexico City, Mexico; 4 Instituto Nacional de Enfermedades Respiratorias, Servicio de Microbiología Clínica, Mexico City, Mexico; Baylor College of Medicine, United States of America

## Abstract

**Background:**

Recycled treated or untreated wastewater represents an important health challenge in developing countries due to potential water related microbiological exposure. Our aim was to assess water quality and health implications in a Mexico City periurban agricultural area.

**Methodology/Principal Findings:**

A longitudinal study in the Xochimilco wetland area was conducted, and 42 sites were randomly selected from 211, including irrigation water canals and effluents of treatment plants. Sample collection took place during rainy and dry seasons (2000–2001). Microbiological parameters (total coliforms, fecal coliforms, streptococci/enterococci, and bacteria other than *Vibrio* grown on TCBS), *Helicobacter pylori*, and physicochemical parameters including trihalomethanes (THM) were determined. Fecal coliforms and fecal streptococci are appropriate indicators of human or animal fecal contamination. Fecal coliform counts surpass Mexican and World Health Organization irrigation water guidelines. Identified microorganisms associated with various pathologies in humans and domestic animals comprise *Escherichia coli*, *Klebsiella* spp., *Salmonella* spp., *Enterobacter* spp., *Enterococcus* spp., and *Pseudomonas* spp; *H. pylori* was also present in the water. An environmental characteristic of the canal system showed high Total Organic Carbon content and relatively low dissolved oxygen concentration; residual chlorine as a disinfection control is not efficient, but THMs do not represent a problem. During the rainy season, temperature and conductivity were higher; in contrast, pH, dissolved oxygen, ammonia, and residual chlorine were lower. This is related with the continuous load of feces from human and animal sources, and to the aquatic systems, which vary seasonally and exhibit evidence of lower water quality in effluents from treatment plants.

**Conclusions/Significance:**

There is a need for improvement of wastewater treatment systems, as well as more efficient monitoring, regulation, and enforcement procedures for wastewater disposal into bodies of water.

## Introduction

Water is an increasingly scarce resource worldwide. One of the most significant changes has been the growth of cities to unprecedented sizes, representing increasing competition associated with the rising demands for water from periurban and rural regions [1]. With regard to population growth, there is an increasing need for food production related with the need of water for irrigation, which implies additional pressure on water resources. With this increase in the scarcity of freshwater resources available to agriculture, the use of urban wastewater in agriculture continues. Wastewater, as both treated and untreated water, is often the only source of water in urban and periurban areas [2]. There is a great difference with respect to treatment capacity: while developed countries treat ca 75% of their discharges, developing nations treat ca <15% of these [3].

The main point is that use of reclaimed water for non-potable uses leaves good quality resources for their most valuable purpose: human consumption [4]. This situation leads to the promotion of irrigation with recycled water [5]. In developing nations where water recycling frequently comprises the response to water shortage, the primary concern lies in risk of microbiological contamination [6]–[8] that can impact public health. Recently, a worldwide evaluation of wastewater irrigated areas presented Mexico as one of the leading water recyclers for irrigation (with approximately 180,000 wastewater irrigated hectares [ha]). These irrigation activities occur with application of water quality regulations based on bacterial indicators and nematode eggs [6], [9], [10].

Although traditional bacterial indicator use is limited for predicting pathogenic microorganisms of fecal origin, lack of assessment of health implications is of greater concern. Other test applications must be considered in order to provide a more reliable evaluation of the health risks imposed by wastewater reuse for irrigation, as well as for potential groundwater system recharge.

In this study, we evaluate water from Xochimilco, a historically relevant agricultural area from Pre-Columbian times that is a periurban agricultural area south of Mexico City. Mexico City is the second megacity worldwide that is facing water scarcity for both human consumption and irrigation. The Xochimilco irrigation canal area covers 207 km and includes rectangular land plots, these an efficient sub-irrigation strategy [11], [12] allowing for one of the most diverse and productive agroecosystems [13], [14].

At present, this area faces several water resources and water quality associated environmental problems, especially from the microbiological perspective [15]–[17]. Since 1945, water has been pumped intensively from Xochimilco to supply Mexico City with the liquid, causing irreversible alterations in regional hydrology due to groundwater pumping, spring capture, wastewater pumping, and urbanization [12]. In 1957, the canals began to be recharged with untreated sewage and treated wastewater [14]. Environmental degradation of this area has been noted, with the presence of high nutrient concentration, as well as high rates of *Escherichia coli* virulence factors and enteric virus within surface water [18]. The Xochimilco agricultural area situation is a strong representation of other periurban regions worldwide, in which rural areas are transformed by urbanization, fostering water shortages. The increasing water shortage drives the unplanned reuse of wastewater. Therefore, the aim of this investigation was to assess water quality and health implications in a periurban agricultural area within Mexico City.

## Methods

### Experimental design

Our sampling scheme was as follows: Sampling sites were spatially selected on a detailed map of Xochimilco scale 1:10,000 (Universidad Autónoma Metropolitana-Gobierno del Distrito Federal, 2000) based on exhaustive determination of perennial canals (with water throughout the year), dividing these into 250 m long sections. This division resulted in a universe of 211 sections to which a unique identification number for each section was assigned. A 42 site sample was obtained through simple randomization of the whole universe; 37 correspond to canal sites and five, to wastewater treatment plant effluents to the canal system. Sampling was performed twice during the 2000–2001 annual cycle, with a single sampling performed during the rainy season (June through September, 2000) and another one time sampling conducted during the dry season (January through May, 2001). The sample size ensured that the binomial 95% confidence interval's (95% CI) upper limit was not >10% when obtaining a 0% point estimate, this assuming that no sample cultured any bacterial indicator (fecal coliforms and fecal streptococci/ enterococci), this in line with current Mexican irrigation water standards and World Health Organization (WHO) recommendations [9], [10], [19].

**Figure 1:**
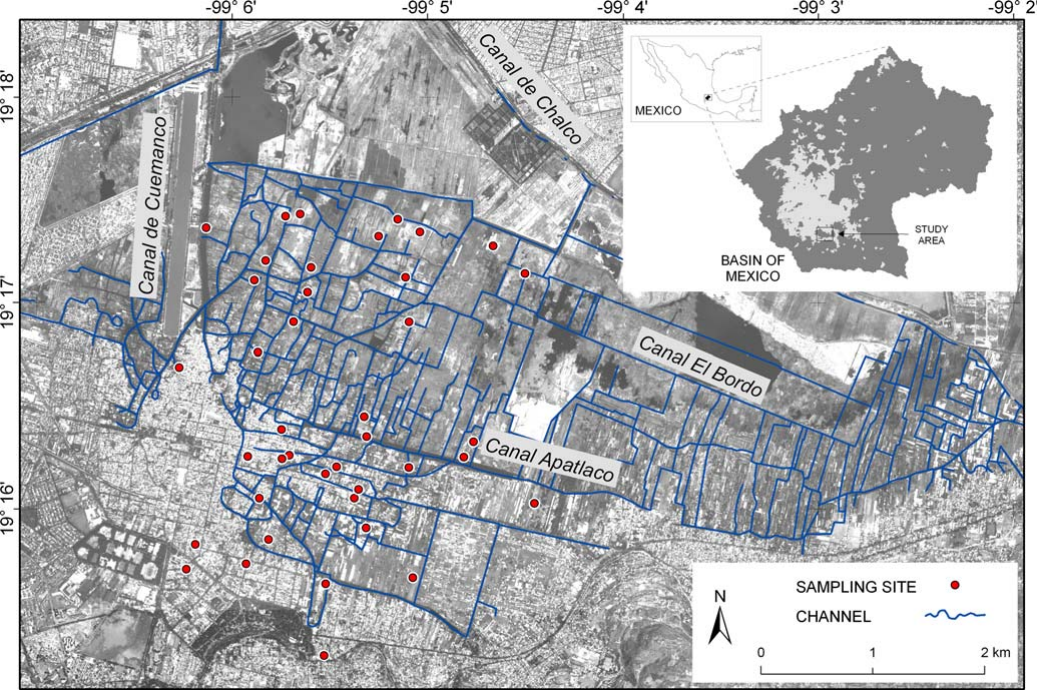
Randomly selected sampling sites in the Xochimilco periurban Mexico City study area.

At each collection point, samples were placed in triplicate flasks for physicochemical (*in situ* measurements, nutrients, and total carbon determinations), as well as bacteriological analyses. Figure 1 shows randomly selected water sampling sites in the Xochimilco aquatic system.

### Bacteriological analyses

One liter samples were collected during selected time points in wide mouth polypropylene sterile flasks for bacteriological analyses. Samples were transported and stored refrigerated (4°C), according to American Public Health Association (APHA) standard procedures [20]. Microbiological samples were processed within 24 h of collection.

Water samples were analyzed following standard membrane filtration procedures for enumeration of four bacterial types, namely total coliforms, fecal coliforms, streptococci/enterococci, and bacteria other than *Vibrio* grown on TCBS. Membrane filters (0.45 µm cellulose acetate, Millipore MF type HA) were placed on a pad with 2.5 ml of m-Endo broth MF for total coliforms, M-FC broth for fecal coliforms, KF Streptococcus agar for streptococci and/or enterococci, and thiosulfate citrate bile salts sucrose agar (TCBS) to isolate bacteria other than *Vibrio*, although this medium was designed for *Vibrio* spp. isolation [20]. Incubation was performed with a WTB Binder brand incubator at 35°C for 24 h for total coliforms, for bacteria other than *Vibrio* grown on TCBS at 35°C for 24–48 h for fecal streptococci and/or enterococci, and at 44.5°C for 24 h for fecal coliforms, according to the APHA [20].

Gram-stain and biochemical tests were used to identify bacteria by a DADE MicroScan, AutoSCAN-4 (DADE International, West Sacramento, CA). Micrococcaceae and Streptococcaceae families that include *Staphylococcus* and *Enterococcus*, as well as those of the Enterobacteriaceae family, were identified.

Positive samples from water filtration isolates were selected based on different morphologies, and five colonies of each were placed on sheep blood 10% agar for both, Gram-positive and Gram-negative bacteria for Gram stain, and McConkey agar for Gram-negative bacteria to differentiate between positive and negative lactose. Then, five colonies for each morphology were selected and identified by negative COMBO22 microplate (Dade-Behring, MicroScan). After the microplate was inoculated with standard bacterial suspension, it was incubated for 18 h at 37°C; subsequently, Vogues-ProsKauer and TDA indol were developed by adding specific reagents. The COMBO22 microplate was read at MicroScan Auto SCAN-4 Dade (West Sacramento, CA, USA).

To identify Gram-positive bacteria, a catalase test was conducted to make a division between positive and negative. The positive were Micrococcaceae and Streptocacceae, and Enterobacteriaceae were negative. Then, the COMBO 12 microplate was used as describe previously.

The results obtained as previously described were expressed as the probability of acceptable identification; only those >85% were considered as true identification. In the case of a lower percentage, additional tests were applied to obtain true bacterial identification.

The presence of *H. pylori* was determined by polymerase chain reaction (PCR) and confirmed by Southern blot hybridization. A 500 ml sample was concentrated by centrifugation at 10,000×*g* for 30 min at 4°C. Sediments were resuspended in 10 ml of 10 mM Tris- HCl (pH 8.0) and 1 mM EDTA (TE), and 1 ml aliquots that were stored at –70°C were used to extract DNA by the guanidium thiocyanate EDTA Sarkosyl EDTA method [21]. Primers and amplification conditions were the same as those used by Mazari *et al.* [22], [23].

PCR products of *16S rRNA* of *H. pylori* were purified utilizing an MER plasmid spin commercial kit (BIO101, La Jolla, CA, USA) from ethidium bromide-stained 3% agarose gel. Products of 110 bp from DNA amplification were cloned in a PCR2.1 TOPO (Kna^R^ Amp^R^) plasmid according to manufacturer instructions (INVITROGEN, Life Technologies). The transformation assay was carried out in *Escherichia coli* XL1-Blue by thermal shock and grown in Luria media with Xgal (artificial galactoside), IPTG (IsopropilB-D- thiogalactoside), and kanamycin. Five selected white clones were cultured in a 5 ml mixture of Luria broth and kanamycin. A pUC18 plasmid was used as clone control with a PCR21 vector without a DNA fragment insert. Plasmid extraction and purification was conducted by alkaline lysis [24]. The PCR extraction products were visualized in 1% agarose gel of 0.5% ethidium bromide-stained TBE buffer.

To determine whether the plasmid contained a *16S rRNA* fragment, a PCRp was used from M13 sequence sites with M13 reverse and forward primers. The PCR cocktail was performed as previously described during 30 cycles. The PCRp were visualized on 2% agarose gel of ethidium bromide-stained 0.5% TBE buffer. To confirm the presence of the DNA fragment insert in the clones, Hp1 and −2 primers were employed with the PCR cocktail and similar conditions as described previously for amplification were followed. A 110-bp band was visualized in agarose gel and stained as mentioned previously to identify the *16S rRNA* nucleotide sequence. The selected clones were sequenced using an ABI PRISM, BigDye Terminator Cycle Sequencing Ready Reaction Kit (PE Applied Biosystems) according to manufacturer instructions. One µl of DNA plasmid was used to sequence in both directions. The electropherogram was obtained using an ABI PRISM 377 DNA Sequencer (PE Applied Biosystems). *H.-pylori cagA* identification was carried out using primers cagA1 (93089): 5′-ATACACCAACGCCTCCAAG-3′, and cagA2 (93261): 5′-TTGTTGCCGCT TTTGCTCTC-3′ as described by Castillo-Rojas *et al*. [25].

### Physicochemical analyses

Five hundred milliliter samples were taken for the following purposes: one for *in situ* parameter determination; one for physicochemical analyses, and one for Total Organic Carbon (TOC); all were analyzed in duplicate. Analysis consisted of temperature, conductivity, and pH measurements from the 500 ml water samples utilizing a portable YSI (model 3500 and serial no. 93J09730). Residual chlorine was analyzed employing an Orion selective electrode (model 9770BN) and calibrated using Orion Potassium Iodated as Chlorine stock solution no. 977007; nitrate was analyzed by Corning electrode (model 476137) and calibrated utilizing standard nitrate solutions prepared from Corning 478163 stock solution, while ammonia was analyzed by Corning electrode (model 487234) and calibrated utilizing standard solutions prepared from Ammonium Standard solution Corning no. 478161. Dissolved oxygen was measured *in situ* using multiparameters (Sension 156, HACH). TOC concentration was determined with a UIC Carbon Analyzer (model CM5012) by the Coulomb metric method (ASTM D 4129-88 and −513-92).

Samples for individual and total trihalomethanes were taken in duplicate in 40 ml amber borosilicate glass vials with screw caps and silicon teflon septum, adding 160 µl 0.2 M sodium sulfite as a reducing agent to samples containing chlorine. Concentration was determined by the *Headspace* technique described by Cancho (personal communication) using a HP 7694 *Headspace* auto-sampler coupled with an HP 5890 Series II Gas Chromatograph with Electron Capture Detector and a DB-624 capillary column 30 m in length, with a 0.25 mm internal diameter and a 2-µm film. Chromatographic conditions were as follows: 200°C injector temperature; 250°C detector temperature; column temperature at 80°C at 1 min, raised by 6°C per min until 140°C was reached and maintained for the final min. Helium was used as a carrier gas at 1 ml/min, and a special mixture of 5% methane in 95% argon was employed for the detector.

### Statistical analysis

Because several physicochemical data and bacterial count distributions were highly skewed, we preferred to show medians (Md) and interquartile intervals (Q1–Q3) as descriptive indexes for central tendency and dispersion, respectively. In addition, for bacterial counts we estimated positivity fraction as quotient of samples with at least one colony forming unit (CFU) of the organism at hand divided by the total number of samples studied; 95% CIs were estimated for medians and point estimates of positivity fractions. The significance of differences between seasons (rainy *vs.* dry) was calculated with the Wilcoxon-Mann-Whitney rank sum test (alpha value, 0.05). In addition, Spearman rank correlation coefficient was calculated among residual chloride, THM, and bacterial counts.

Although the fecal coliform/fecal streptococci ratio [26] has been questioned due to the differential die-off kinetics of the two bacterial groups, it is considered valid for recent fecal contamination (24 h). The ratio was applied as a rapid approach for fecal origin tracking, which requires minimum expertise to show a general tendency of the possible contamination source [27]–[29].

## Results

**Figure 2:**
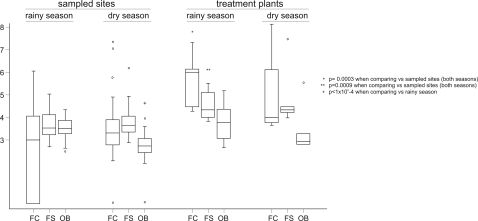
Box and whiskers plots of results of microbiological analyses of Xochimilco area irrigation by season and site. Bacterial counts are shown in a log_10_ scale; empty circles denote outliers.

Results of the microbiological analyses of the Xochimilco area irrigation water samples by season and site (Figure 2), demonstrate a clearly higher amount of fecal coliforms and streptococci in treatment plants during both seasons, as well as a lower bacterial count for other bacteria during the dry season at the sampled sites. The fecal coliform/fecal streptococcus ratio was significantly higher in treatment plants than at sampled sites (medians: 3.84 vs. 0.36; *p* = 0.0041). Analysis by positivity rate, not shown in the figure, revealed a higher value for fecal coliforms during dry season at the sampled sites (62.2 vs. 97.3%; *p*<1×10^−4^), as well as for the *Helicobacter pylori* 16S rRNA strain during dry *vs.* rainy season, and also at the sampled sites (27.8 *vs.* 55.9%; *p* = 0.028).

Due to interference with the growth of high total coliform densities on Endo medium and the uncertain statistical analyses, these results were not considered for general analysis. It is clear that total coliforms used as water quality indicators have serious limitations, especially as indicators in tropical areas, as mentioned in the Discussion section.

Bacteria isolated from water samples following standard membrane filtration procedures were plated, purified, and identified to ascertain the actual pathogens present in treatment plant effluents that supply water to the canals (the main water source), as well as those in irrigation water. Isolated and identified genus and species are shown in Tables 1 and 2.

**Table 1:** Microorganisms identified in water samples from treatment-plant effluents that supply water to the Xochimilco canal system

Microorganisms isolated	Rainy season (*n* = 5)	Dry season (*n* = 5)
Total coliforms		
*Escherichia coli*	12	12
*Klebsiella pneumoniae*	3	3
*Enterobacter cloacae*	-	4
*Enterobacter agglomerans*	1	-
*Salmonella* spp.	1	-
*Hafnia alvei*	-	1
Fecal coliforms		
*Escherichia coli*	20	13
*Enterobacter cloacae*	2	5
*Enterobacter aerogenes*	2	-
*Klebsiella pneumoniae*	1	3
Streptococcaceae		
*Enterococcus* spp.	36	68
*Streptococcus viridans*	-	1
Micrococcaceae		
*Staphylococcus* spp.	-	29
Non-fermenters		
*Pseudomonas aeruginosa*	-	1
Gram-negative non-fermenter bacteria	8	-
Total	86	140

**Table 2:** Microorganisms identified in Xochimilco-canal water samples

Isolated microorganisms	Rainy season (*n* = 37)	Dry season (*n* = 37)
Total coliforms		
*Escherichia coli*	24	63
*Klebsiella pneumoniae*	15	45
*Klebsiella oxytoca*	3	14
*Enterobacter cloacae*	10	34
*Enterobacter aerogenes*	3	-
*Citrobacter freundii*	1	1
*Proteus mirabilis*	1	-
*Providencia rettgeri*	-	4
*Serratia odorifera*	9	-
*Salmonella arizona*	2	-
*Hafnia alvei*	-	6
Fecal coliforms		
*Escherichia coli*	57	69
*Enterobacter cloacae*	1	-
*Enterobacter aerogenes*	1	-
*Klebsiella pneumoniae*	4	11
*Citrobacter freundii*	1	-
*Edwarsiella tarda*	3	-
*Hafnia alvei*	1	-
*Salmonella typhi*	-	1
Streptococcaceae		
*Enterococcus* spp.	238	257
*Streptococcus viridans*	3	2
Micrococcaceae		
*Staphylococcus* spp.	4	36
*Micrococcus* spp.	4	-
Non-fermenters		
*Pseudomonas aeruginosa*	2	7
*Pseudomonas* spp.	9	-
*Acinetobacter* spp.	4	-
Gram-negative non-fermenter bacteria	44	58
Total	444	608

Surface water that recharges Xochimilco area canals is obtained from three wastewater treatment plants. Analyses performed at five treatment plant outlets show the presence of fecal coliform and fecal streptococci and/or enterococci (Table 1); these are considered human and warm blooded animal fecal contamination indicators that survive in the environment and that can last for several months. In samples obtained from the canal system (Table 2), it was possible to identify enterobacteria genus not isolated from treatment plants. This can be due to canals receiving not only treated water, but also discharges from irregular human settlements and cattle raising, which contain a greater diversity of microorganisms.

Physicochemical parameters by season and site are shown in Table 3. Significant differences can be appreciated in the majority of parameters at sampled sites according to season. During the rainy season, temperature and conductivity were higher. On the other hand, in the same season pH, dissolved oxygen, ammonia, and residual chlorine were lower as compared with dry season. Among other parameters, it is noteworthy that during the rainy season there is an increase in chloroform, bromodichloromethane, dibromochloromethane, and total THM (the latter four not shown in Table). Residual chlorine concentration is usually lower than recommended levels for microorganism disinfection or inactivation. At sampled sites, residual chlorine demonstrated a positive association with microbiological indicators as follows: fecal coliforms (*r* = 0.51; *p* = 0.0000), and fecal streptococci (*r* = 0.42; *p* = 0.0002). A negative correlation was present only for other enterobacteria; this correlation was also significant (*r* = –0.27; *p* = 0.0210). Additionally, only four samples were higher than the recommended residual chlorine concentration for water (<0.2 mg/l).

**Table 3:** Physicochemical parameters for Xochimilco area irrigation canal water

	Sampled sites		Treatment plants	
	Rainy season (*n* = 37)	Dry season (*n* = 37)	Rainy season (*n* = 5)	Dry season *(n* = 5)
Variable	Md (95% CI)†	Md (95% CI)	Md (95% CI)	Md (95% CI)
temperature (°C)	20.4 (20–20.9)	15.3 (15.0-16.2)****	19.7 (18.1–20)	18 (16.9–18.9)
Conductivity (S/cm)	905 (711-–988.8)	719 (699.2–727.9)*	683 (530–941)	745 (650–935)
pH (no units)	7.3 (7.04–7.36)	7.66 (7.38–8.18)***	6.86 (6.74–7)	7.28 (6.69–7.37)
dissolved oxygen (mg/l)	2 (1.41–3.78)	6 (3.41–9.32)****	5.4 (3.4–6.8)	5.8 (3.8–9)
no_3_ (mg/l)	17.3 (14.52–28.63)	11.97 (6.76–17.42)	15.72 (0.9–43.68)	4.21 (0.6–366.08)
nh_4_ (mg/l)	0.68 (0.222–1.567)	4.03 (2.58–6.10)****	1.0001 (0.83–2.19)	7.55 (0.02–222.65)
residual chlorine (mg/l)	0.0007 (0.00021-0.01177)	0.011 (0.0070–0.0189)**	0.05 (0.00002–0.07)	0.012 (0–0-235)
Total organic carbon (mg/l)	45.15 (26.12–100.06)	32.5 (15.22–49.77)	55.76 (7.88–126.95)	97.6 (6.8–393.56)

† Md : Median; CI : Confidence interval.

* *P = *0.012 when comparing against rainy season (sampled sites). Wilcoxon-Mann-Whitney ranksum test;

** *P* = 0.0035 when comparing against rainy season (sampled sites). Wilcoxon-Mann-Whitney ranksum test;

*** *P = *0.0002 when comparing against rainy season (sampled sites). Wilcoxon-Mann-Whitney ranksum test;

**** *P*<1×10^−4^ when comparing against rainy season (sampled sites). Wilcoxon-Mann-Whitney ranksum test.

## Discussion

Seasonal differences in the Basin of Mexico, where Xochimilco is located as a highland wetland, its location 2,240 m above sea level and between the tropics and its receiving higher radiation rates than at sea level are important factors. From the climatic perspective, the seasons can be sub-divided in dry (November–April) and rainy (May–October). During the latter, the area is maintained with rainfall combined with untreated wastewater from irregular settlements, while during the dry season the system is mainly maintained by treated water flowing from three wastewater treatment plants and also combined with untreated wastewater.

Based on the microbiological, data there is a significant difference between the rainy and dry seasons; fecal coliforms data are consistent with our previous studies [18], [30], [31]. Fecal streptococci/enterococci and bacteria other than *Vibrio* growing in TCBS are present throughout the year. The orders of magnitude in which the various bacterial groups are reported agree with the range within which bacteria are found in untreated domestic wastewater [32].

A variety of microorganisms was found in the Xochimilco agricultural area, such as Enterobacteriaceae, *Escherichia coli, Enterobacter cloacae*, *Klebsiella pneumoniae*, *K. oxytoca, Citrobacter freundii*, and *Salmonella* spp., as well as non-fermenters such as *Pseudomonas* spp. and *Acinetobacter* spp. Some species are not native to the natural environment and may represent exogenous microorganisms, further indicating a human or animal fecal source. The observed patterns of irregular urban area settlements and animals such as cows or sheep grazing in some areas provide suggestive evidence of the non-native microorganisms.

The most prevalent bacteria were *Enterococcus* spp. and *E. coli*, which were three times more prevalent during the rainy season, as compared with five times the prevalence of *Enterococcus* during the sampling period's dry season. *Enterococcus* spp. can be suggested as an alternative water quality indicator based on the prevalence observed.

As mentioned by Mara et al. [33], no pathogen identification has been carried out in Mexican epidemiological studies; thus, disease could have been due to more than one pathogen. Because water reclaimed and reused for irrigation in developing countries has a greater and more diverse microorganism load than that in developed countries that have better treatment and control processes, it is necessary to identify microorganisms and to quantify them in order to relate this to the health threat.

In our study, the diversity of microorganisms in treated water is lower than in canal water used for irrigation, with some specific genus of fecal origin in both waters, such as *E. coli*, *Klebsiella*, *Enterobacter*, *Pseudomonas*, and *Citrobacter* [34]. Although the treatment process is not very efficient, it appears to be reducing the diversity of bacteria, with a further bacterial supply from untreated contributions to the aquatic system. Greater control of irregular settlements, associated with additional social and economic limitations, is obviously required.

As shown in Table 2, two relevant species from the public health perspective that cause gastrointestinal diseases requiring antibiotic treatment are *Salmonella arizona* and *S. typhi*. The former is reported during the rainy season and the latter, during the dry season.

Regarding the use of some groups as microbial water quality indicators of water, some stress has been reported as an interference with total coliform growth on Endo medium. Several non coliform bacteria have been shown to inhibit coliform bacteria growth, and coliform bacteria have been shown to produce non-sheen colonies on membrane filter on m-Endo medium and are often referred to as a background count [35]. Several non *Pseudomonas aeruginosa* caused significant reductions utilizing Endo medium for *E. coli* sheen colony counts when the former were present at high densities [35].

Dominating strains composed of background colonies on m-Endo agar may inhibit coliform growth at incubation temperatures of 44.5°C [36]. A resuscitation step has been proposed for fecal coliform enumeration in tropical waters [37]; notwithstanding this, we attempted this step in a groundwater quality evaluation in Mexico City [38] and observed no significant differences with the method suggested by APHA [20].

Based on temperature and background colony growth, detection of fecal rather than total coliforms using m-FC agar has been mentioned as reasonable for fecal pollution microbiological monitoring in eutrophic surface water in temperate regions [36].

On the other hand, the m-FC culture method has been reported as inadequate for fecal coliform enumeration in subtropical water samples. In addition to *E. coli*, some non-*E. coli* thermophilic coliform isolates can also grow on plates at 44.5°C, these considered false positive errors [39].

Therefore, more specific methods are needed for obtaining a realistic view of microbial water quality in tropical and subtropical areas. Identification of bacteria as performed in this work is a costly and labor intensive method that is not applicable in routine analyses. An alternative and rapid method would be application of molecular techniques. But such methods imply modernization of monitoring laboratories as well as the updating of technical operators, additionally implying an investment in infrastructure and personnel training, these urgently required for facing environmental problems, especially in the field of water management.

Mexican irrigation water and fresh water organism quality standards [9], [40], [41] and WHO guidelines [10], [19] specify ≤1,000 CFU/100 ml as the fecal coliform limit for acceptable irrigation water for crops likely to be eaten uncooked and for sport fields and parks: this limit is surpassed in areas of Xochimilco. Although fecal streptococci/enterococci presence and levels are not considered among Mexican, U.S. Environmental Protection Agency (EPA), or WHO irrigation or aquatic life protection water standards, these bacteria were targeted because the group has been suggested as an alternative indicator [28].

The fecal coliform/fecal streptococci index was applied [26], [27], [29]. The index suggests >2 as contamination from a human source, as shown in 29% of samples; this is slightly more frequent in the rainy season (34%), as compared with the dry season at (24%) (*p* = not significant). Two thirds of fecal samples appeared to be of animal origin. The contamination source must be further studied and confirmed molecularly; however, the data presented provides a rapid and low cost method for determining the contamination's origin.

We previously reported *H. pylori* detection in 20% of groundwater samples and cagA gene presence in 40% of positive samples [17], while in this study we detected *H. pylori* in 44% and cagA gene in 14% of positive samples. Differences in light, temperature, and TOC conditions in ground and surface water appear not to be determinant for *H. pylori* counts and distribution.

With respect to the canal system's environmental characteristics, one common characteristic for both dry and rainy seasons showed high TOC content and relatively low dissolved oxygen concentration (especially during the rainy season). This means that the system has an excessive load of organic matter that can be attributed to years of receiving urban run-off without adequate treatment, as well as nutrients from non point due to fertilizer application for agricultural activities. Nitrates are found at a relatively high concentration, but within the range of domestic raw wastewater. This means that irrigation water is of low quality, based on some physicochemical parameters.

Broad regulations exist in Mexico for agricultural irrigation water. These regulations take into account solids, Biochemical Oxygen Demand (BOD), total nitrogen and phosphorous, and heavy metals. No specific forms of nitrogen measured in this study are regulated, nor are TOC. Biological specific limits are provided for bacteria and helminths.

Levine and Asano [42] consider that water quality parameters relevant to water reclamation and reuse such as nitrogen fall into an approximate range in treated water of 10–30 mg/l and regarding phosphorous, of 0.1–30 mg/l. Free chlorine is also considered in concentrations <1.0 mg/l [43], as well as pH within the range of 6.0–9.0 for irrigation or 6.5–9.5 for protection of aquatic life [41].

Wastewater, in addition to its use as a fertilizer, supplies nitrogen compound load to the aquatic system, providing an environment excellent for the flourishing of microorganisms. Thus, we conducted several analyses involving physicochemical parameters that exhibited no significant correlation with microbiological determinations. The characteristics of organics in reclaimed water area were measured with non specific parameters such as TOC; the latter is also a measurement of organic matter in Xochimilco water. TOC showed a continuous high concentration throughout the year [7], and concentrations can double or nearly triple during the rainy season, a fact that can be attributed to soil washing by precipitation that on average can reach 1,500 mm annually in the Basin of Mexico's southwestern area.

Highly degraded organic carbon load is related with high nutrient concentrations, in this case, nitrogen forms. Ammonia usually originates from organic matter reduction [44]; in Xochimilco, ammonia predominates during the dry season when wastewater contributed to the system is not diluted by precipitation with relatively better dissolved oxygen concentrations, *vs.* during the rainy season. In general, dissolved oxygen is quite variable throughout the year; this is considered an adequate oxygen concentration only during the dry season, in aquatic media, and for organism preservation.

Although according to Mexican legislation for nitrogen forms this would be good irrigation water based on limnological characteristics, there are high ammonia and nitrate amounts as compared with natural water bodies, rendering the system hypereutrophic. What occurs in sediment and irrigated soils plays a very important role in the fate and transport of TOC and nutrients, an issue needing to be addressed because important microbiological activity must take place in this medium.

As reported for eutrophication assessment in tropical lakes [45], the Xochimilco area receives a significant nitrogen and phosphorus load (not reported) from bovine cattle. In developing countries, the contribution of sewage and wastewater as point sources, as well as animal raising without adequate outlet control, represent extra loads for aquatic systems, the majority of these without treatment, used subsequently for irrigation, and not taken into account as potential risks. This has health implications at local and national levels, in addition to economic implications for exportation of food not complying with international standards.

This case study was conducted in a highly populated area, a story that repeats itself in developing countries and that must be taken into account. Regarding the reclaimed water residual organic fraction, attention must be paid to trace organics such as endocrine disrupting compounds and pharmaceutically active compounds including antibiotics [7], [46], an issue that is not addressed but nonetheless a health concern with unidentified health effects.

Because sediments act as reservoirs or autochthonous and allochthonous particular matter added or produced in the water column [44] and may represent the habitat for numerous organisms, they are extremely important to study. In addition to microbiological impacts, there are technical and health challenges that must be evaluated when utilizing untreated and reclaimed water for irrigation purposes, an emerging field yet to be investigated.
